# Comparison of Soil Phosphorus Tests for Assessing Plant Availability of Phosphorus in an Ultisol Amended with Water-Soluble and Phosphate Rock Sources

**DOI:** 10.1100/tsw.2010.174

**Published:** 2010-09-01

**Authors:** E. W. Gikonyo, A. R. Zaharah, M. M. Hanafi, A. R. Anuar

**Affiliations:** ^1^ Institute of Tropical Agriculture, Faculty of Agriculture, Universiti Putra Malaysia, Serdang, Selangor, Malaysia; ^2^ Department of Land Management, Faculty of Agriculture, Universiti Putra Malaysia, Serdang, Selangor, Malaysia

**Keywords:** phosphate rock, phosphorus bioavailability, phosphorus soil tests calibration

## Abstract

The effectiveness of different soil tests in assessing soil phosphorus (P) in soils amended with phosphate rocks (PRs) is uncertain. We evaluated the effects of triple superphosphate (TSP) and PRs on extractable P by conventional soil tests (Mehlich 3 [Meh3] and Bray-1 [B1]) and a nonconventional test (iron oxide–impregnated paper, strip). Extracted amounts of P were in the order: Meh3 >B1 > strip. All the tests were significantly correlated (*p* = 0.001). Acidic reagents extracted more P from TSP than PRs, while the strip removed equal amounts from the two sources. The P removed by the three tests was related significantly to dry matter yield (DMY), but only in the first harvest, except for B1. Established critical P levels (CPLs) differed for TSP and PRs. In PR-fertilized soils, CPLs were 27, 17, and 12 mg P kg^-1^soil for Meh3, B1, and strip, respectively, and 42, 31, and 12 mg P kg^-1^ soil, respectively, in TSP-fertilized soils. Thus, the strip resulted in a common CPL for TSP and PRs (12 mg P kg^-1^ soil). This method can be used effectively in soils where integrated nutrient sources have been used, but there is need to establish CPLs for different crops. For cost-effective fertilizer P recommendations based on conventional soil tests, there is a need to conduct separate calibrations for TSP- and PR-fertilized soils.

### INTRODUCTION

Phosphorus (P) deficiency is a major constraint to crop production in tropical and subtropical acid soils, and P fertilizers are required to sustain optimum plant growth[1]. In Malaysia, phosphate rocks (PRs) have been used almost exclusively as the P source for crops. Interest in PRs as direct-application fertilizers stems mainly from the fact that PR is usually the cheapest fertilizer per kilogram of P. In addition, PRs are considered environmentally benign and sometimes used in many other countries where the organic farming practice is catching on. PR or fertilizer recommendations for cost-effective crop production should be based on soil test results.

Conventional soil tests were developed to aid fertilizer recommendations in soils fertilized with water-soluble fertilizers, but are frequently not effective with slowly water-soluble fertilizers or PRs[2]. Generally, in soils treated with PRs, alkaline extractants, such as Olsen and Colwell tests, underestimate the soil P status, while acid extractants, such as Mehlich-1, Bray-1, Bray-2, and citric acid tests, tend to overestimate the soil P status[3,4]. However, Mehlich 3 is considered to be a universal extractant (multinutrient extractant) for a wide range of soils, including calcareous soils[5].

An alternative approach to conventional chemical extraction of P is the use of ion sinks, such as iron strips. Ion sinks have given promising results when applied to soils with diverse chemical and physical properties[6,7]. While the chemical extractants enhance desorption of P from the labile fraction, they also change the ionic strength and pH drastically, and do not necessarily mimic conditions in actual soil solution. On the other hand, ion sinks lead to relatively little change in soil and more closely simulate the absorption by plant roots. Ion sinks adsorb P onto the sink surface and interact minimally with the soil, whereas chemical extraction results in soil reaction with the extractant. Consequently, the question to answer from this study is: How do the conventional and nonconventional ion sink methods compare in PR-fertilized soils? To date, limited trials have been conducted on the applicability of conventional and nonconventional methodologies to estimate P bioavailability in PR-fertilized soils. In addition, the critical P levels (CPLs) for the different P sources/methods in PR-fertilized soils are unknown.

The objectives of this investigation were to (1) study the efficacy of available P determination by two conventional soil tests and the ion/sink nonconventional method test from soils treated with PR sources relative to a water-soluble P fertilizer; (2) relate sequentially harvested *Setaria* dry matter yield (DMY), P uptake, and relative yields from PR- and TSP-treated soils to P extracted by the three soil test methods; and (3) establish CPLs for the three soil test methods.

### MATERIALS AND METHODS

#### Experimental Site and Phosphate Sources

The field trial was conducted at the Puchong experimental farm, Universiti Putra Malaysia (UPM) in Serdang, Selangor. The soil is classified as Bungor soil series, an Ultisol (Typic Kandiudult, clayey, kaolinitic, isohyperthermic).

Phosphate extractability from PR- relative to TSP-amended soils was assessed for Mehlich-3 (Meh3), Bray-1 (B1), and iron oxide–impregnated paper strip (strip). Two PRs were used consisting of (1) Gafsa PR (GPR) and (2) Christmas Island PR (CIPR), the most prevalent PR in Malaysia (41% of all PRs).

#### Experimental Design and Layout

*Setaria* grass *(Setaria anceps Stapf*. Cv. Kazungula) was used as the test crop because it has widespread occurrence, can survive in low soil fertility, and shows good response to nitrogen (N) and P application coupled with its suitability for sequential harvesting. Experimental treatments were factorial combinations consisting of 20 treatments ([nil + (3 P fertilizer sources × 3 P rates)] × 2 levels of manure) with three replications arranged in a randomized complete block design (RCBD). The P sources (TSP, GPR, and CIPR) were applied at 100, 200, and 300 kg P $ha^{-1}$ alone (sole) or combined with cattle manure at zero (0) or 20 t $ha^{-1}$.

Fertilizer rates were based on previous “P recapitalization” studies in tropical acid soils[8]. Phosphatic fertilizers and manure (treatments) were applied once (2 weeks before planting) on plots measuring 1.5 × 1.5 $m^2$. At planting and after every cutting, all plots received a basal application of N and K at 60 kg N $ha^{-1}$ as urea and 100 kg K $ha^{-1}$as muriate of potash (MOP), respectively. The grass was established from seeds by broadcasting 0.3-g seeds (>80% germination) along five rows per plot at 30-cm inter-row width in all the plots. The plots were weeded by hand as deemed necessary. Grass harvesting was done by hand cutting with a sickle at 3–5 cm above the soil surface from the three inner rows, discarding harvest from a 0.25-m distance from each plot boundary (harvest area = 0.9 $m^2$). The harvesting interval was 2 months for a period of 14 months.

The harvested grass was dried in a draught-blown oven at 70˚C until no further change in weight and DMY was recorded. A subsample of the dried grass was ground and analyzed for nutrient uptake by ashing in the muffle furnace at 300˚C for 4 h and then 550˚C for another 4 h. After cooling, 2 mL concentrated HCl was added to the crucible and heated to dryness. Then, 10 mL of 20% $HNO_3$ was added and heated on a water bath for 1 h. The solution was then filtered and P, K, Ca, Mg, and Zn in solution were measured using inductively coupled argon plasma spectrophotometry (Model: Thermo Elemental IRIS Advantage).

### Soil Sampling and Analysis

Initial sampling for baseline soil information was done before the treatments were applied. Composite soil samples were taken at soil depths of 0–15 and 15–30 cm before the experiment commenced and at the end of the experiment, while only topsoil samples were collected at every harvest. Subsamples were taken from a mixture of six to eight soil cores per plot, taken in a zig-zag fashion using a 6-cm diameter soil auger. The soils were air dried and ground with a mortar and pestle to pass through a 2-mm-size sieve.

Available P was analyzed using the strip, B1, and Meh3 extracting solutions. The strip papers were prepared according to Menon et al.[6]. They were prepared by passing filter paper No. 541 swiftly through a 10% $FeCl_3$ solution. The paper was air dried and then treated with ammonium vapor to oxidize the iron chloride on the paper to iron oxide. The papers were then air dried, cut into 10- × 2-cm strips, and stored for subsequent use. Phosphorus was extracted from soil by shaking 1 g of soil sample and one paper strip in 40 mL of 0.02 M KCl end-over-end for 16 h. The strip was removed from the solution, rinsed free of adhering soil particles, and air dried. Phosphorus retained on the strip was eluted from the strip by shaking it end-over-end with 40 mL of 0.1 M $H_2SO_4$ for 2 h[9].

Meh3 P was extracted at 1:20, soil/solution ratio with 5 min of shaking[10]. The extractant comprised 0.2 M $CH_3COOH$ + 0.25 M $NH_4NO_3$ + 0.015 M $NH_4F$ + 0.013 M $HNO_3$ + 0.001 M EDTA. After filtration, P was read from the supernatant by colorimetry. B1 P was extracted at 1:10 soil/solution ratio and shaken by hand for 5 min, followed by P determination in the supernatant colorimetrically[11]. Extractant comprised 0.03 M $NH_4F$ + 0.025 M HCl[12].

### Statistical Analysis and Calculations

All data were analyzed using the Statistical Analysis System[13]. Duncan's multiple range test (DMRT) was used for mean separation within each harvest. Analysis of variance (ANOVA) was performed on the combined data using the PROC MIXED procedure. Harvests and blocks were considered random variables in the model, while fixed effects were the treatments. Statistical significance was assessed at the 0.05 level. The Cate-Nelson graphical method[14] was used to determine the CPLs.

## RESULTS

### Soil and Fertilizer Characteristics

Soil and fertilizer characteristics are presented in Tables 1 and 2, respectively. The soil was strongly acid (pH 3.9 in KCl) with moderately high P fixing capacity (P sorption maximum = 769 mg $kg^{-1}$soil). Characterization of the fertilizers indicated that TSP had the highest P content, while GPR had the lowest among the three fertilizers. However, GPR was more soluble than CIPR (Table 2).

**TABLE 1**
**Characteristics of the Top- and Subsoil**

| Parameter | Topsoil | Subsoil |
|---|---|---|
| pH water (1:2.5) | 4.5 | 4.5 |
| pH KCl (1:2.5) | 3.9 | 3.9 |
| Total C (g $kg^{-1}$) | 30 | 20 |
| Total P (mg P $kg^{-1}$ soil) | 280 | N/D† |
| P sorption maximum (mg P $kg^{-1}$ soil) | 769 | 833 |
| Available P (mg P $kg^{-1}$ soil) by: | | |
| B1 | 4.9 | 2.6 |
| Olsen | 5.2 | 2.6 |
| Pi strip | 0.6 | 0.4 |
| $Al_2O_3$ (g $kg^{-1}$) | 15.2 | 15.2 |
| $Fe_2O_3$ (g $kg^{-1}$) | 30.8 | 31.6 |
| CEC (cmol [+] $kg^{-1}$) | 5.4 | N/D† |

† Not determined.

**TABLE 2**
**Characteristics of the Fertilizers Used**

| Composition | Fertilizers (g $kg^{-1}$) | | |
|---|---|---|---|
| | TSP | GPR | CIPR |
| P | 204 | 125 | 140 |
| $P_2O_5$ | 462 | 289 | 323 |
| CaO | 192 | 455 | 420 |
| $CaO/P_2O_5$ | 0.4 | 1.57 | 1.3 |
| $Fe_2O_3$ | 6 | 4 | 31 |
| $Al_2O_3$ | 16 | 9 | 107 |
| MgO | 29 | 9 | 23 |
| Solubility in water* | 380 | 0.2 | 0.1 |
| Solubility in 2% citric acid* | 430 | 100 | 87 |
| Solubility in 2% formic acid* | 407 | 110 | 66 |

* Solubility based on total $P_2O_5$.

### Extractable P

All the three test methods extracted more P from the fertilized plots than the nonfertilized plots and they exhibited wide extractable P ranges; Meh3 P exhibited the widest range (0.9–101.1 mg P $kg^{-1}$ soil), while B1 P closely followed (1.3–98.6 mg P $kg^{-1}$ soil), and the narrowest was strip P (0.8–53.4 mg P $kg^{-1}$ soil) (Table 3A). On average, Meh3 extracted 21.3 mg P $kg^{-1}$soil (28% more P than B1), while the strip on average extracted 56% P compared to B1.

When amounts of P extracted were partitioned according to the P sources, it was observed that Meh3 and B1 extracted significantly different amounts of P from PR- and TSP-treated soils ($p < 0.0001$). Alternatively, the strip extracted nonsignificantly different amounts of P from the PR and TSP treatments, implying that the strip was insensitive to the P sources. A closer observation revealed that Meh3 extracted

**TABLE 3A**
**Amounts of P Extracted by the Three Soil Test Methods**

| Variable | P Extraction Method | | |
|---|---|---|---|
| | Meh3 | Strip | B1 |
| Number of samples (N) | 294 | 294 | 294 |
| Maximum (mg P $kg^{-1}$ soil) | 101.1 | 53.4 | 98.6 |
| Minimum (mg P $kg^{-1}$ soil) | 0.9 | 0.8 | 1.3 |
| Mean (mg P $kg^{-1}$ soil) | 21.3 | 9.3 | 16.6 |
| S.E ± (mg P $kg^{-1}$ soil) | 1.06 | 0.49 | 0.97 |

relatively similar amounts of P from CIPR and GPR (on average, 16 mg P $kg^{-1}$ soil); in contrast, it extracted more than 200% P from TSP relative to the PRs (Table 3B). Similarly, B1 extracted relatively similar amounts from the two PRs (on average, 10–11 mg P $kg^{-1}$ soil) and from TSP-treated soil, 31.2 mg P $kg^{-1}$ soil (≈300% P relative to the PRs) as shown in Table 3B. Comparing Meh3 and B1 P revealed that the former extracts about 50% more P from the PRs than B1, while it extracts only 13% more P from TSP than B1 (Table 3B).

**TABLE 3B**
**Amounts of P Extracted by the Three Soil Test Methods According to P Sources**

| P Sources | Extractable P (mg P $kg^{-1}$ soil) | | |
|---|---|---|---|
| | Meh3 | Strip | B1 |
| CIPR | 16.0 ± 0.98 | 10.2 ± 0.94 | 9.7 ± 0.61 |
| GPR | 15.5 ± 1.07 | 7.7 ± 0.54 | 11.4 ± 0.79 |
| TSP | 35.3 ± 2.64 | 11.9 ± 1.11 | 31.2 ± 2.48 |

Combined analysis of extractable P over time for the seven samplings (at every grass harvest) for each soil test method revealed that both Meh3 and B1 P were significantly influenced by P source ($p$ = 0.0001) and P rate ($p$ = 0.0001), and generally decreased with time. The amounts of Meh3 and B1 P increased with increasing fertilizer application rates. For example, at the beginning, control plots gave an extractable P of 5.5 and 3.8 mg P $kg^{-1}$soil for Meh3 and B1, respectively. The highest amounts extracted were 101.1 and 76.6 mg P $kg^{-1}$ for Meh3 and B1, respectively, from 300 kg P $ha^{-1}$ applied as TSP. Application of 100 and 300 kg P $ha^{-1}$ increased Meh3 P by more than 100 and 500% of the control plot, respectively (Fig. 1). Among the three P sources used, the lowest Meh3 P was extracted from CIPR-treated soils (≈12 mg P $kg^{-1}$ soil), while about 2- and 1.3-fold were extracted from TSP and GPR, respectively, in accordance to their solubility, but the two PRs were not significantly different.

### Correlation of the Test Methods

Correlation of the three soil test methods using 294 soil samples revealed they were highly correlated. The acidic extractants (Meh3 vs. B1) exhibited the highest correlation coefficient (r = 0.84**, n = 94), while the correlation coefficients for Meh3 vs. strip, and B1 vs. strip, were about 50% lower than that of the conventional tests (r = 0.43** and 0.44**, respectively) as shown in Fig. 2.

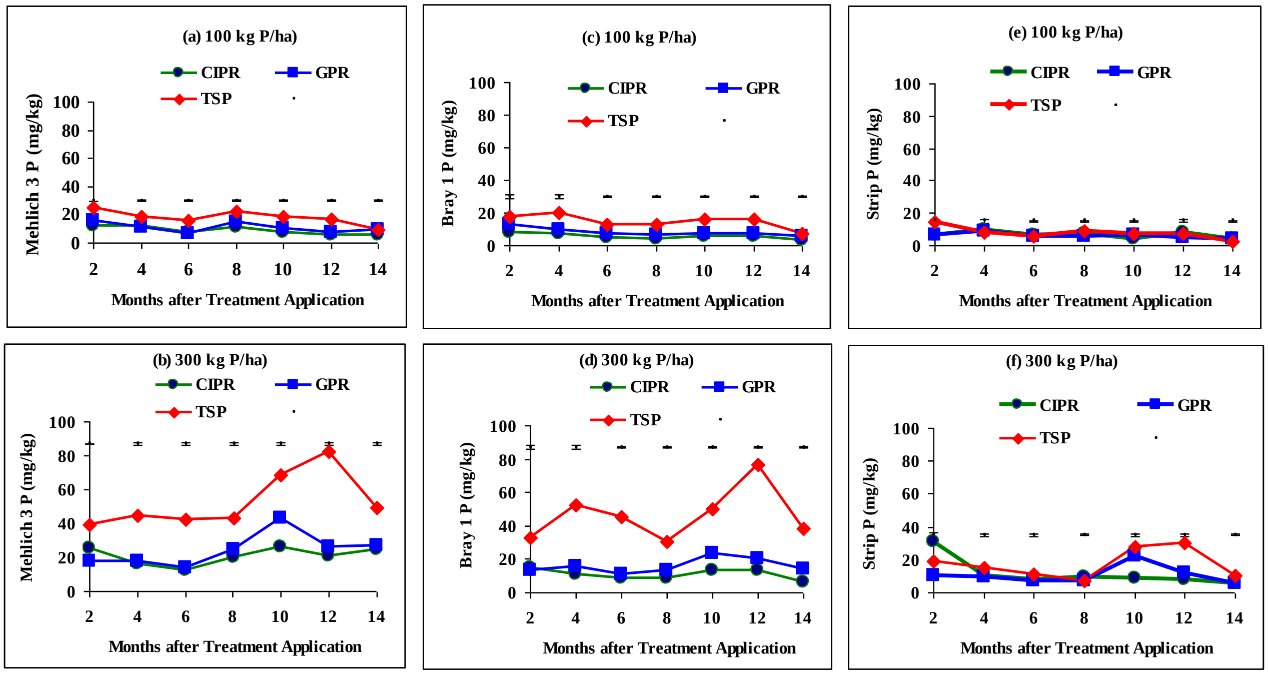

**FIGURE 1**. Phosphorus extracted from the three P sources at 100 and 300 kg P ha$^{-1}$ by (a,b) Meh3, (c,d) B1, and (e,f) strip. Note that the bars denote S.E. of the means and the data points for TSP at 300 kg P ha$^{-1}$, months 10 and 12 were considered outliers.

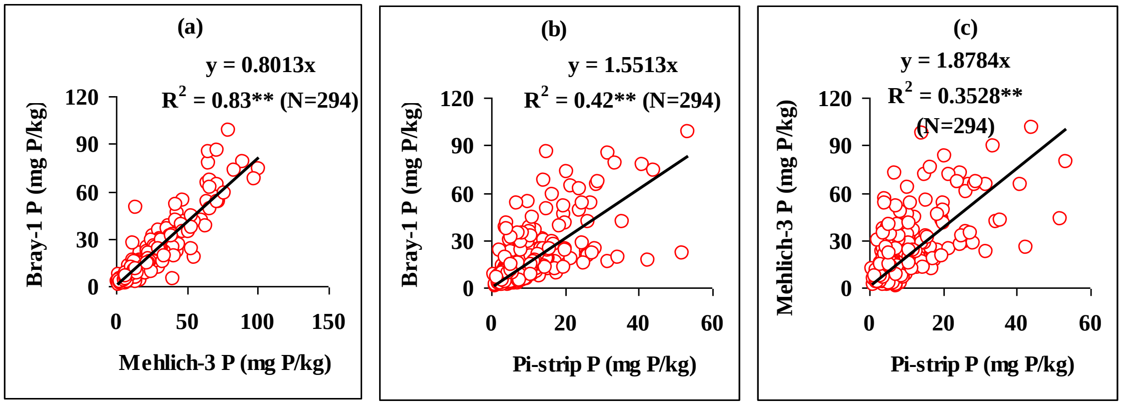

**FIGURE 2.** A comparison of the three soil test P methods; (a) B1 vs. Meh3, (b) B1 vs. strip, and (c) Meh3 vs. strip.

### Relationship between Extractable P vs. DMY

Regression of all the DMY data for the seven harvests on P extracted from the seven soil samplings (at every harvest) by the three soil test methods resulted in highly significant relationships for DMY against all the three methods with very similar regression coefficients, which were very low; 0.12, 0.11, and 0.11 for B1, Meh3, and strip, respectively (Figs. 3A–3C). When data were partitioned by the P sources, both Meh3 and B1 P gave two regression lines: one for TSP and the other for PRs. However, the amount of maximum yields attained for TSP and PRs were relatively similar, but they were attained at different P concentrations for the two P sources. Maximum yields were attained at a higher P level in TSP than in PRs (Figs. 3A and B). In contrast, strip data partitioning by P sources resulted in almost a single line for both TSP and PRs (Fig. 3C).

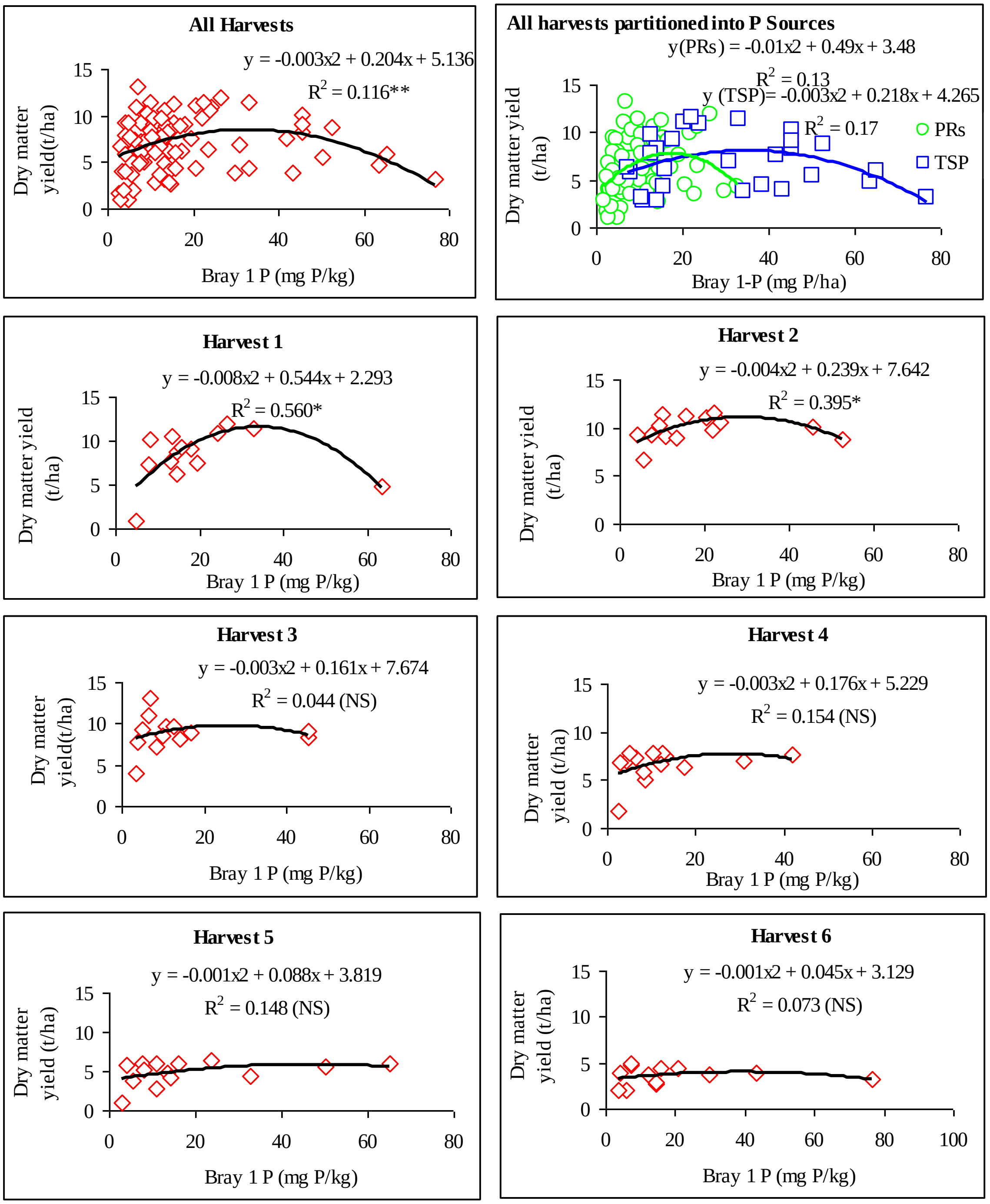

**FIGURE 3A**. Relationship between B1 extractable P and *Setaria* DMY for all harvests, all harvests partitioned into different P sources, and harvests 1–6. NB: * and ** denote significance at 5 and 1%, respectively. NS denotes not significant.

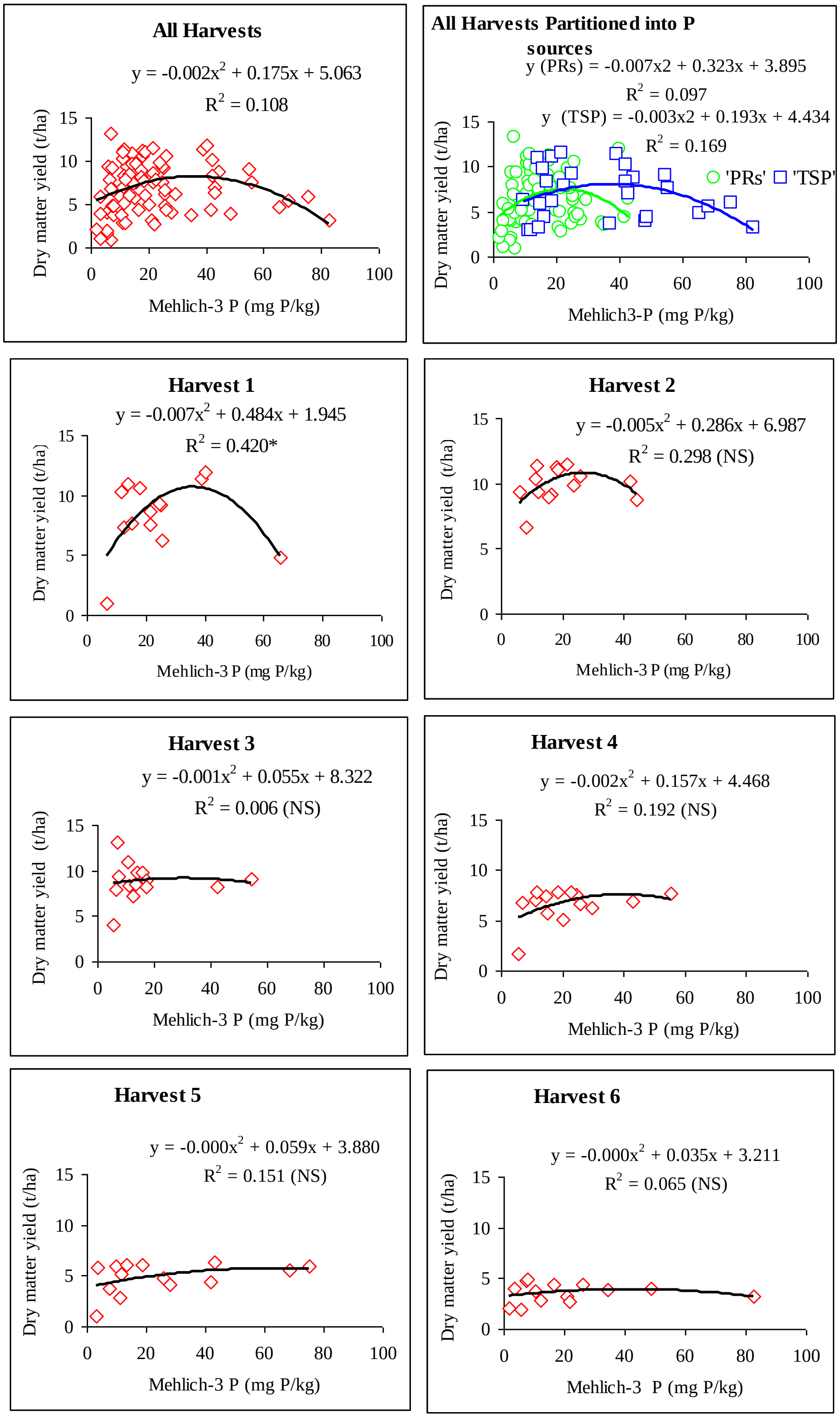

**FIGURE 3B**. Relationship between Meh3 extractable P and *Setaria* DMY for all harvests, all harvests partitioned into different P sources, and harvests 1–6. NB: * and ** denote significance at 5 and 1%, respectively. NS denotes not significant.

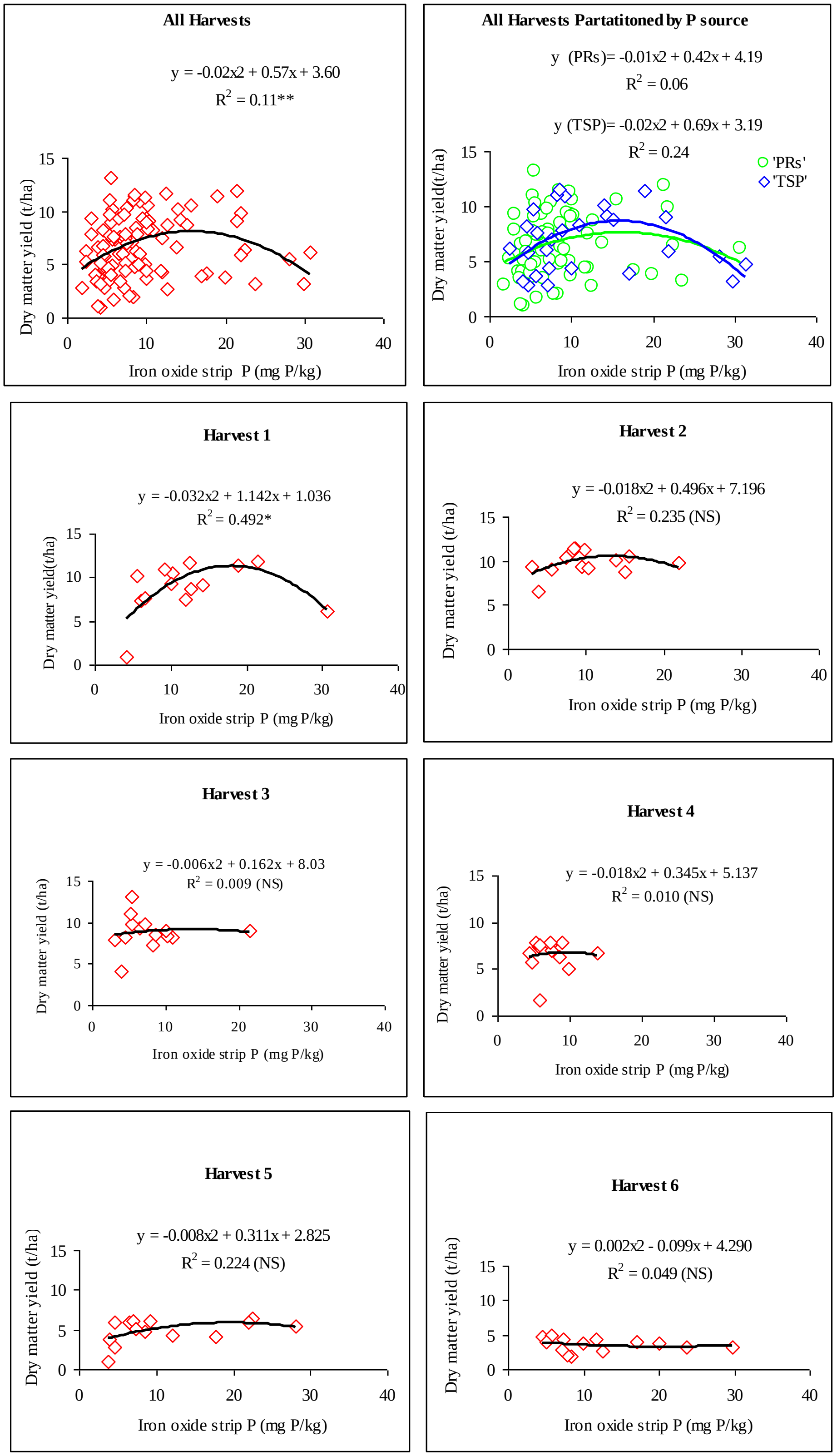

**FIGURE 3C.** Relationship between strip extractable P and *Setaria* DMY for all harvests, all harvests partitioned into different P sources, and harvests 1–6. NB: * and ** denote significance at 5 and 1%, respectively. NS denotes not significant.

Further data partitioning by harvests indicated that only the first harvest resulted in significant relationships of DMY against extractable P for the three soil tests, while all other subsequent harvests showed nonsignificant relationships (Figs. 3A–3C). The only exception was Harvest 2 in B1 P, which also indicated some significance. Thus, DMY was only explained by extractable P in the first harvest after P application, but in subsequent harvests could not be explained by extractable P.

### Relationship between Extractable P by the Three Soil Test Methods vs. P Uptake by *Setaria*

Regression coefficients of P uptake by *Setaria* vs. extractable P by the three soil tests and CPLs established for the three soils are presented in Table 4. When all the data of P uptake (from seven harvests) were related to extractable P (from soils sampled at every harvest) by the three soil test methods, the relationships were much better than the relationships with DMY for the three soil tests. Phosphorus uptake exhibited quadratic relationships with regression coefficients about double those of DMY vs. extractable P. The $R^2$ were 0.26, 0.21, and 0.21 for B1, Meh3, and strip P, respectively (Table 4). When data were partitioned by harvests, Meh3 and B1 P gave similar results, whereby P uptake and extractable P were significantly related in five out of seven harvests. On the other hand, relationship of P uptake against the strip P resulted in only three out of seven harvests exhibiting significant relationships (Table 4).

**TABLE 4**
**Regression Coefficients for B1, Meh3, and Strip Extractable P on *Setaria* P Uptake**

| Harvests | B1 | Meh3 | Strip |
|---|---|---|---|
| | Regression Coefficient ($R^2$) | | |
| All harvests | 0.26** | 0.21** | 0.21** |
| 1 | 0.55* | 0.33 | 0.47* |
| 2 | 0.30 | 0.29 | 0.21 |
| 3 | 0.43* | 0.40* | 0.39 |
| 4 | 0.52* | 0.56* | 0.13 |
| 5 | 0.57* | 0.64** | 0.72** |
| 6 | 0.59* | 0.54* | 0.13 |
| 7 | 0.38 | 0.27 | 0.62** |

*Note*: * and ** denote significance at 5 and 1% levels, respectively.

### Critical P levels for Meh3, B1, and Strip Test Methods

Meh3 and B1 exhibited different critical P levels for TSP- and PR-fertilized soils, while the strip exhibited a common CPL for the two P sources. In PR-fertilized soils, the CPLs were 27, 17, and 12 mg P $kg^{-1}$soil compared to 42, 31, and 11 mg P $kg^{-1}$ soil in TSP-fertilized soils for Meh3, B1, and strip tests, respectively (Fig. 4). In TSP-fertilized soils, most outliers were found in the lower right quadrant and almost none in the upper left quadrant (Fig. 4 ia, iia, and iiia) of the Cate-Nelson plots for the three soil tests. On the other hand, in the PR plots, there were more outliers in the upper right quadrant than the left lower quadrant for the three soil tests (Fig. 4 ib, iib, and iiib). Thus, the strip soil test resulted to common CPLs for TSP and PRs.

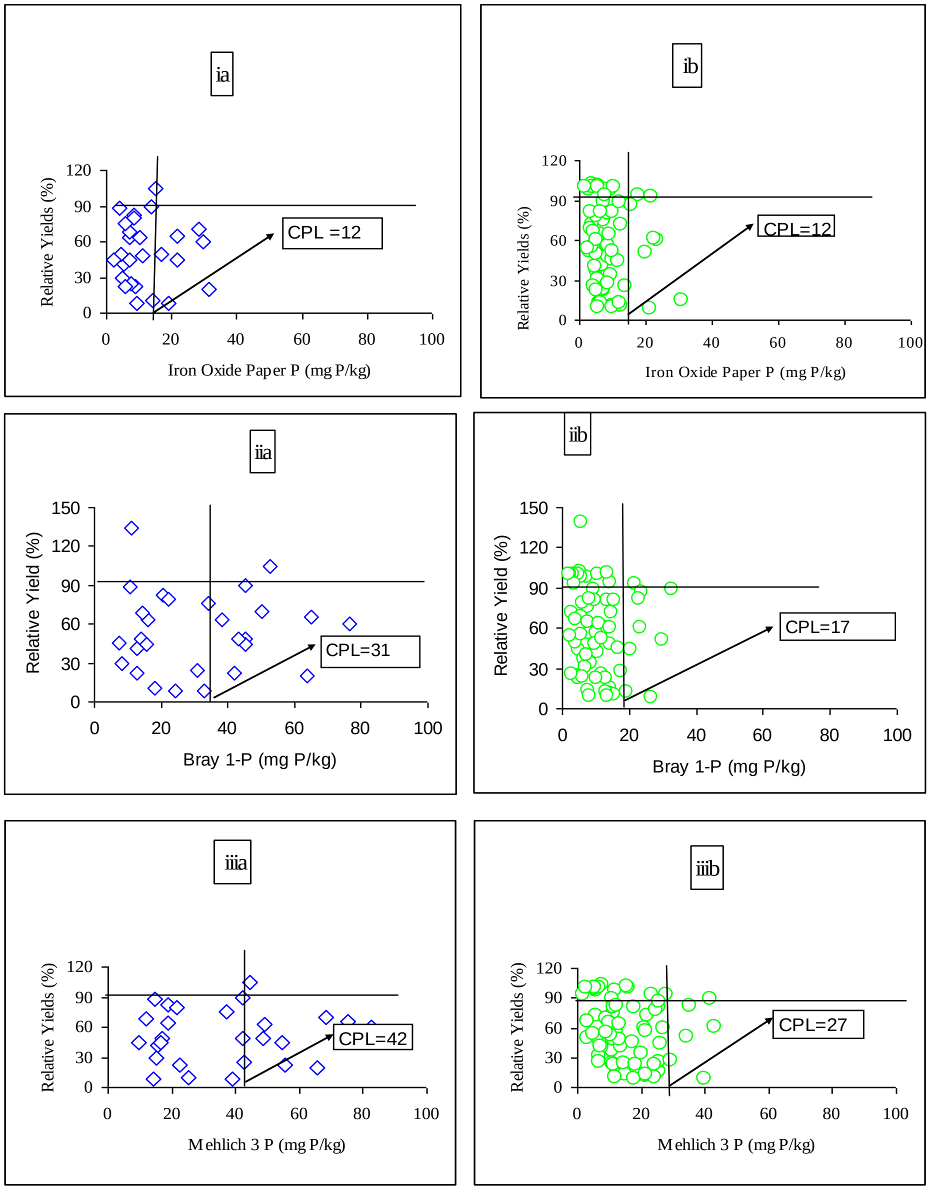

**FIGURE 4.** CPLs for the P sources (a) TSP- and (b) PR-fertilized soils for the test methods: (i) strip, (ii) B1, and (iii) Meh3. Arrow: denotes the CPL established.

## DISCUSSION

### P Extractability

As expected, the extracted P varied widely with the test method used because each extractant removed different portions of inorganic P. Meh3 and B1 extracted the highest P from both the nonfertilized and fertilized soils. This is because both Meh3 and B1 are acidic extractants that could partly remove water-insoluble Ca-P and Mg-P compounds that the sorption/desorption process involved in the iron oxide strip method cannot remove. This is particularly true for the undissolved PR and, hence, resulted in lower amounts of P extracted by the strip relative to the other two methods. Meh3 extracted the highest P because the solution is highly buffered compared to B1, as demonstrated by Mallarino[15] in one soil that was high in pH and $CaCO_3$ content. Similarly, Kleiman et al.[16] reported higher Meh3 and B1 than strip P removed from 24 soils sampled across the U.S., varying widely in chemical and physical characteristics with soil pH ranging from 4.2 to 8.6. Nonetheless, other workers reported higher strip P than B1 and Meh3[17,18,19].

The general observation was that B1 or Meh3 always extracted more P than the strip P from the lower pH soils (pH < 5.5). Accordingly, the soils tested in this work, with low pH (4.6) and high iron and aluminium oxides (15.2 and 30.8 g $kg^{-1}$ soil, respectively) showed higher extractable Meh 3 and B1 P more than the strip P. The opposite holds true that in higher pH soils, the strip extracts more P than B1 and sometimes Meh3. The different amounts of P removed by the different reagents relative to one another reflect the differences in both the P-extracting abilities of different reagents and soil properties associated with P availability. Meh3 was developed for use in widely differing soils (acidic and calcareous), while B1 was developed for acidic to neutral soils and will extract the highest amounts of P from soils with pH < 5[18], but will extract less P than even the strip in soils with pH > 7.0[19]. The lower extractability of P by the B1 extractant in calcareous soils is usually attributed to neutralization of the acid-extracting solution by $CaCO_3$ and precipitation of the fluorine to calcium fluoride.

Amounts of extractable P from the different P sources were in the order TSP > GPR = CIPR for the two acidic reagents (Meh3 and B1), consistent with the solubility of the fertilizers with an exception of the two PRs, which gave similar amounts of extractable P in the field experiment despite their solubility difference. This observation was in contrast to findings from the incubation experiment for the same PRs, where GPR gave statistically higher extractable P than CIPR (data not shown).

The enhanced solubility of the CIPR in the field giving rise to comparable Meh3 and B1 P in GPR is probably due to the removal of PR dissolution products, such as Ca, by plant uptake and leaching, and P mainly by plant uptake and fixation by the soil components, all of which have been reported to enhance PR dissolution[20]. However, due to the stronger buffering capacity of Meh3, it extracted more P from the PRs than B1. Similar results of lower P extraction by B1 than Meh3 from PR-treated soils were reported by Indiati and Neri[17]. The fact that different amounts of P were extracted from the PRs and TSP (soluble P sources), coupled with the fact that conventional soil tests were developed and calibrated for use with water-soluble P fertilizers, implies there is a need to calibrate the methods for use with nonsoluble P sources This could be done from the expensive field trials or from correlation studies.

The strip method was insensitive to the P sources and is, therefore, suitable for use with any of the P sources. This ion sink procedure is considered to have potential use in widely ranging soils and fertilizers, water-soluble as well as -insoluble P[17]. It was also found to be an effective method for assessing available P in calcareous soils[19] and, consequently, may be suitable in soils where PRs have been used over time, such as in Malaysia.

The high correlation between the two acidic reagents and relatively lower correlations between the conventional methods and the strip method is a reflection of the disparity in the sink method and the acid-extracting methods[21]. Other workers also reported strong relationships between Meh3 and B1, and similarly high correlations between the two acidic extractants and the strip[16,17].

### Relationship between Extractable P and Plant Parameters

The low regression coefficients obtained between extractable P by the three methods and DMY of *Setaria* grass are explained by the fact that Meh3 and B1 have been shown not to extract the more recalcitrant P fractions, such as the sodium hydroxide organic P (NaOH-Po), which to a great extent influenced DMY, besides the sodium hydrogen bicarbonate inorganic P ($NaHCO_3$-Pi) and hydrochloric acid P (HCl) fractions[22]. This explained the low regression coefficient between B1 P, Meh3, and DMY. The same authors also showed that P uptake was better related to extractable P than DMY, because the former was mainly determined by $NaHCO_3$-Pi and residual P, while the latter was determined mainly by NaOH-Po. Consequently, since both Meh3 and B1 extracted mainly the $NaHCO_3$-Pi, P uptake was better related to extractable P by the two methods than DMY.

The nonsignificant relationship of extractable P with DMY in subsequent harvests after the first one could probably be explained by the fact that, with time, P continued to be converted to more recalcitrant forms that were not extractable by the two reagents (Meh3 and B1), but influenced DMY, such as NaOH-Po and residual P[23].

### Critical P levels for the Different Soil Test Methods in TSP- and PR-Fertilized Soils

Meh3 extractant resulted in the highest CPLs than B1 P, which in turn was also higher than strip P in both TSP- and PR-fertilized soils. They followed the P extractability trend (Meh3 > B1 > strip). If the soil tests are highly correlated, the CPLs for the newer soil tests could be established by correlation with the older soil tests, as was done for Meh3 when it was adopted in the northeast region of the U.S. The tentative CPLs for Meh3, of 37 and 42 mg P kg$^{-1}$ soil in PR- and TSP-treated soils, are within the 30–50 mg P kg$^{-1}$ soil CPLs reported from various states in the U.S.[24,25], and the B1 CPLs, of 31 and 17 mg P kg$^{-1}$ soil in TSP- and PR-treated soils, and were also close to the CPL of 30 mg P kg$^{-1}$ soil reported by the same authors.

The CPLs are known to vary with different extractants, soil characteristics, and type of crop[26]. Researchers working on soils and crop response data collected in 70 sites across 31 districts for 5 years in Kenya showed that the CPLs for maize were15 and 10 mg P kg$^{-1}$ soil for low and high carbon soils (< or > 1.5%), respectively, for modified Olsen P[26]. Meh1 CPL for maize was 30 mg P kg$^{-1}$ soil for all the soils and it was similar for sorghum, but for common bean, cabbage, and potato, it was ≈50 mg P kg$^{-1}$ soil.

Different CPLs established for TSP and PRs implies that the decision to apply fertilizer or not in a soil will vary depending on the history of P fertilizers used. If a soluble fertilizer has been applied in the past, the decision to apply fertilizer will be at a higher extractable P value than if PRs have been used.

Limited calibrations have been done on soil tests for use of PRs and, hence, CPLs for TSP have been used to guide fertilizer recommendations consequently, making an error of applying P where it is unnecessary, despite the high cost of the fertilizers. The other difficulty will be in deciding which CPLs to use in the field where both the water-soluble (TSP) and sparingly soluble (PRs) have been used. The strip test method will be the most ideal for testing soils regardless of the fertilizer history, since it showed no sensitivity to P sources as evidenced by the common CPLs for TSP- and PR-treated soils. No CPLs for the strip method were found in the literature for comparison.

### CONCLUSIONS

The results of this study demonstrated the need for different P calibrations for soil tests in PR- and TSP-fertilized soils. The different CPLs (mg P kg$^{-1}$ soil) for Meh3, B1, and strip P in soils established were as follows: Meh3, 27 (PR) and 42 (TSP); B1, 17 (PR) and 31 (TSP); and strip, 12 (both PR and TSP). The differences between the CPLs in TSP and PRs for Meh3 and B1 are quite substantial. Thus, when TSP-established CPLs are used for fertilizer recommendation in PR-fertilized soils, the probability of applying P in PR-fertilized soils, while P is unnecessary or gives no yield response, is quite high. This will be in the fields/plots with P levels between 27 and 41 for Meh3, and 17 and 30 mg P kg$^{-1}$ soil for B1.

Considering the increasing use of PRs in the plantations and upcoming organic farming, the erroneous application of P may translate to a reasonably high economic effect. Consequently, establishment of CPLs in PR-fertilized soils for different crops/soils cannot be overemphasized. The use of fertilizer mixtures of TSP and PRs is common because, sometimes, to achieve faster responses to P, a “starter dose” of TSP is usually recommended in combination with PRs. On the other hand, there have been switches from TSP to PRs or vice versa, hence demonstrating the importance of a soil test method insensitive to the different P sources.

The current study demonstrated that the strip soil test method is insensitive to the P sources. Therefore, further research to establish CPLs for different crops and soils for the strip method is recommended.

### ACKNOWLEDGMENTS

We are grateful to the Institute of Tropical Agriculture for offering a postdoctoral fellowship and to the Third World Organization of Women in Sciences (TWOWS) for the Ph.D. fellowship offered to the first author, Esther W. Gikonyo. Finally, thanks to the staff of the Department of Land Management, Faculty of Agriculture, Universiti Putra Malaysia. where the research was conducted.
